# In vitro evaluation of the influence of titanium nitride coating on the retention force between components of two-part abutments

**DOI:** 10.1186/s12903-021-01636-7

**Published:** 2021-06-02

**Authors:** Nadine Freifrau von Maltzahn, Jan Holstermann, Meike Stiesch, Philipp Kohorst

**Affiliations:** 1 Department of Prosthetic Dentistry and Biomedical Materials Science, Hannover Medical School, Carl-Neuberg-Str. 1, 30625 Hannover, Germany; 2 Crüsemannallee 78, 28213 Bremen, Germany; 3 Lilienthaler Heerstr. 261, 28357 Bremen, Germany

**Keywords:** Two-part abutments, Titanium nitride coating, Bond adhesion, Surface treatments, Extractor test

## Abstract

**Background:**

Two-part abutments are typically made up of a base composed of titanium and a ceramic build-up. The long-term outcomes are affected by the mechanical durability. The purpose of the present investigation was to evaluate and compare the retention force of two-part abutment systems with titanium or titanium nitride bases—as fixed with zirconia components and with various surface treatments.

**Methods:**

A total of 60 two-part abutments were investigated—with a titanium base (n = 30) or titanium nitride coated bases (n = 30) and bonded with zirconia ceramic build-ups. The bonding surfaces were treated with aluminium oxide blasting, with an average particle size of 110 µm. The titanium bases were then pretreated with Alloy Primer or Clearfil Ceramic Primer. The ceramic build-ups were only treated with Clearfil Ceramic Primer. For twenty test specimens, no chemical pretreatment was performed. Test specimens were classified into six groups in accordance with the pretreatment (A–F; n = 10). A resin-based luting agent was employed to attach the two parts. Specimens were then subjected to artificial thermal aging (10^4^ cycles with 5 °C/55 °C). The retention force between the two parts was then investigated with a pull-off test. The findings were analyzed by ANOVA statistics. Fracture patterns were examined by electron microscopy.

**Results:**

In the absence of primer*,* titanium nitride coated bases gave significantly greater retention forces than other samples (*p* < 0.05). Chemical preconditioning with silane coupling agents did not effect on the retention force of coated bases.

**Conclusions:**

The results of the current study suggested that modifying metal surfaces by coating the base with titanium nitride not only has esthetic and biological advantages, but also enhances the mechanical properties of the adhesive bond of two-part abutments.

## Background

If implant-prosthetic components are to be esthetically pleasing and satisfying, it appears to be critical to optimize the material and shade of the abutments. Two types of implant abutments have been used: standardized titanium (stock) abutments and customized abutments. Most abutments are now produced by computer-aided design (CAD) and manufacture (CAM), as adjusted to the individual anatomic circumstances of each oral cavity [1, 2]. To assure desirable mechanical properties, most abutments have been pure titanium. On the other hand, this has the disadvantage that it leads to grey discoloration of surrounding tissue, especially in the anterior region—where the gingiva is often thin [3, 4]. For this reason, ceramic abutments have been used to achieve better esthetic results. Dental ceramics may be attractive in appearance, with excellent biocompatibility, and high mechanical stability [5, 6]. Nevertheless, they have the disadvantage that they fracture more readily than titanium abutments [7]. Another critical point is that abutments composed of dental ceramics cannot be produced with the same fitting accuracy as titanium ones, so that there may be marginal misfit—with negative consequences such as micromotion, screw loosening and increasing microgap between implant and abutment [8–11]. For this reason, two-part abutment systems could be a good alternative, as they are composed of an individually fabricated, esthetic ceramic superstructure fixed with a resin-based luting agent onto a standardized stable titanium base [12, 13]. On the other hand, the weakness of these two-part systems is that the adhesive connection between the two components can influence the clinical success over the time. Several examinations have proved that surface pretreatment can influence the retention force between components of two-part abutment systems [13–16]. In particular, air abrasion may enhance the retention forces between the two components of two-part abutments [13, 14]. Pure titanium is commonly used for bases of two-part abutments. On the other hand, several published articles have shown promising findings with pretreatment of conventional titanium for one-part abutments systems—such as coatings with titanium nitride (TiN) [17, 18]. For example, Ferrari et al. compared abutments of titanium, titanium nitride and zirconia in a clinical trial. As judged by mechanical failure, optimal results were obtained after three years for titanium and titanium nitride abutments [17]. Chung et al. investigated the retention force between titanium-aluminum nitride coating and dental ceramics and showed that the flexural bond strength between the two materials can be increased by coating [19]. To our knowledge, there is no published study which evaluates the influence of titanium nitride coating on the retention force to dental ceramic in two-part abutment systems—particularly as this approach might lead to two-part abutments which are both esthetically attractive and mechanically stable. Therefore, it would be interesting if titanium nitride coating not only provides an esthetically pleasing result with good mechanical properties for one-part abutments, but also if it can optimize the retention force in two-part abutments.

The aim of the current investigation was to evaluate the retention forces of titanium nitride-coated bases in comparison to conventional titanium bases and to analyze the effect of different surface modifications on the retention force between the two components of two-part abutments. Before retention force testing, all specimens were subjected to artificial thermal aging to imitate the conditions of the oral cavity. The test hypothesis was that the retention force of titanium nitride coated bases to ceramic build-ups is comparable to standardized titanium bases with similar surface modifications.

## Methods

The surfaces of a total of 60 two-part abutments with titanium (n = 30) or titanium nitride coated bases (n = 30) and build-ups of zirconia ceramic were differentially modified and cemented with adhesive. The retention force of the two components in all 60 test specimens were investigated after thermal aging between 5 and 55 °C, in order to simulate long-term aging results. Microscopic analyses were then performed.

The sample size was not based on a power analysis, but on previous studies already published on this topic.

### Test specimens

**Fig. 1:**
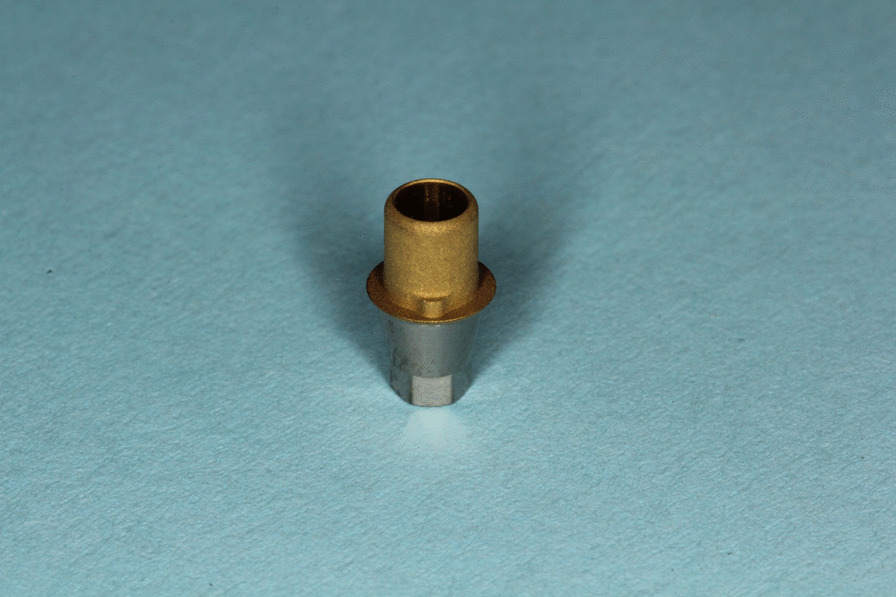
Prefabricated titanium base of test specimen coated with titanium nitride

**Fig. 2:**
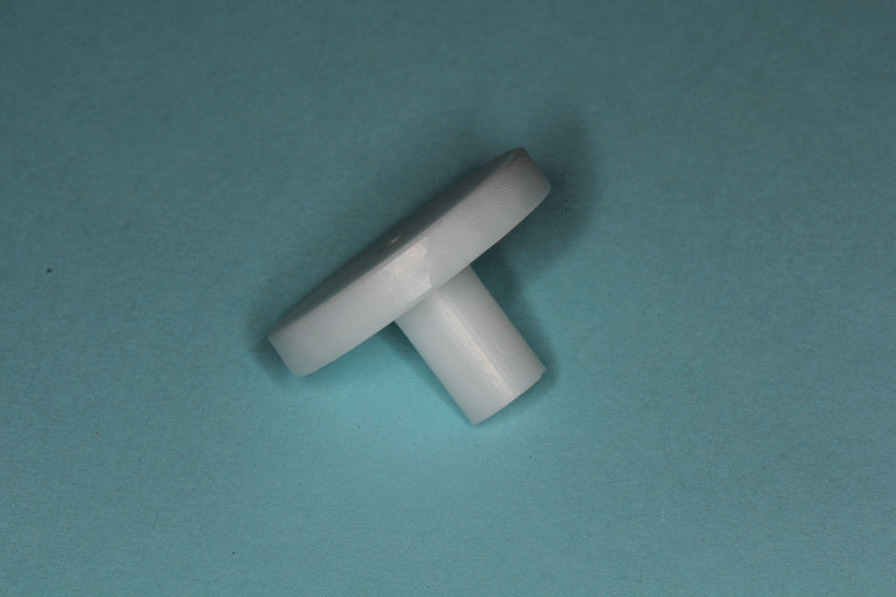
Zirconia coping of test specimen

**Fig. 3:**
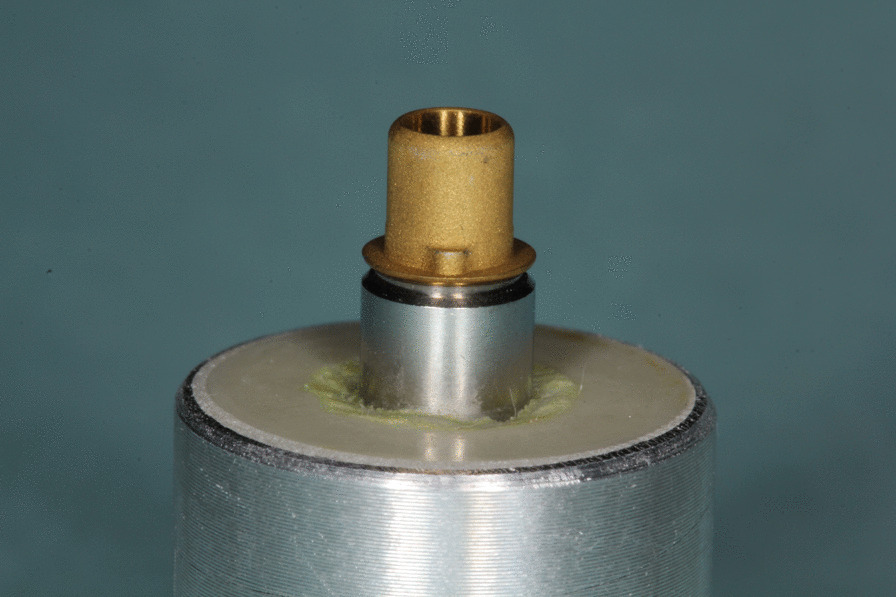
Test specimen embedded in polyurethane

All specimens consisted of standardized titanium bases or titanium nitride coated bases (S 1020, Medentika, Hügelsheim, Germany) and zirconia ceramic build-ups, produced by CAD/CAM (CADSPEED GmbH, Nienhagen, Germany) from a presintered Y-TZP material (Zirkon Biostar, Siladent, Goslar, Germany) and with a customized design for the subsequent pull-off tensile test [20]. The thickness of coating was measured with 1.8 µm and was conducted by the company Medentika. The luting gap was adjusted to 30 µm. The cylindrical base of titanium had a height of 7.8 mm and an upper aperture of 3.4 mm (Fig. 1). The build-ups of zirconia had a height of 11 mm, a bore diameter of 3.5 mm and a major diameter of 5 mm (Fig. 2). All samples were enclosed into a pedestal of polyurethane (AlphaDie MF, Schütz Dental Group, Rosbach, Germany) (Fig. 3). Then bases were fixed into laboratory implants with a torque of 35 Ncm. Surfaces of bases and copings were then pretreated and the two components were attached with the help of a resin-based luting agent (Panavia F 2.0, Kuraray Europe GmbH, Hattersheim am Main, Germany), as described below.

### Test procedures

#### Surface treatment

All surface modifications are shown in Table 1. The bonding areas of 30 pure titanium bases (group A-C) and all zirconia build-ups were blasted with aluminum oxide powder (Shera,Lemförde, Germany) with an average particle size of 110 µm and at a distance of approximately 5 mm to the surface area and a pressure of 0.2 MPa for 30 s. The titanium nitride coated bases (n = 30) were blasted by alumina before they were coated. Then components were cleaned up in an acetone bath and blown dry. Bonding areas were treated with different adhesive systems. The activating agent Alloy Primer (Kuraray Europe, Frankfurt am Main, Germany) was used for titanium bases of groups B (uncoated titanium) and E (titanium nitride coated). According to the manufacturer´s instructions, this adhesion promoter is a metal conditioning agent indicated for bonding between dental metals and resin-based materials. Alloy Primer is based on acetone, 10-methacryloyloxydecyl dihydrogen phosphate and 6-(4-vinylbenzyl-n-propyl) amino-1,3,5-triazine-2,4-dithione. The surfaces of bases of groups C (uncoated titanium) and F (titanium nitride coated) as well as the ceramic build-ups of groups B, C, E and F were modified with the silane coupling agent Clearfil Ceramic Primer (Kuraray Europe). This activating primer was based on 3-methacryloxypropyl trimethoxy silane, 10-methacryloxydecyl dihydrogen phosphate, and ethanol. The indication of Clearfil Ceramic Primer is described for surface treatments of ceramics, hybrid ceramics, or composite resins. The components of groups A and D were not chemically pretreated.

**Table 1:** Surface modifications and fixture resin used for different test groups

Group	Titanium bases (n = 30)		Zirconia build-ups (n = 30)		Luting Agent
	Mechanical	Chemical	Mechanical	Chemical	
A (n = 10)	110 µm Al_2_O_3_		110 µm Al_2_O_3_		Panavia F 2.0
B (n = 10)	110 µm Al_2_O_3_	Alloy Primer	110 µm Al_2_O_3_	Clearfil Ceramic Primer	Panavia F 2.0
C (n = 10)	110 µm Al_2_O_3_	Clearfil Ceramic Primer	110 µm Al_2_O_3_	Clearfil Ceramic Primer	Panavia F 2.0

Group	Titanium nitride coated bases (n = 30)		Zirconia build-ups (n = 30)		Luting agent
	Mechanical	Chemical	Mechanical	Chemical	
D (n = 10)	110 µm Al_2_O_3_ before coating		110 µm Al_2_O_3_		Panavia F 2.0
E (n = 10)	110 µm Al_2_O_3_ before coating	Alloy Primer	110 µm Al_2_O_3_	Clearfil Ceramic Primer	Panavia F 2.0
F (n = 10)	110 µm Al_2_O_3_ before coating	Clearfil Ceramic Primer	110 µm Al_2_O_3_	Clearfil Ceramic Primer	Panavia F 2.0

#### Fixation of the components

According to the manufacturers' instructions, the dual-hardening fixation resin Panavia F 2.0 (Panavia F2.0, Kuraray Europe GmbH, Frankfurt am Main, Germany) was applied for all specimens. The outer surfaces areas of bases and the inner surfaces of the ceramic part were exposed to a constant film of luting agent (Table 1). The two components were pressed together manually with a force of 5 kilos for 5 s—controlled by a scale. Surpluses were removed from the whole specimens and light-cured for a time period of 90 s to initiate self-polymerization (Uni XS, Heraeus Kulzer, Hanau, Germany). To ensure the ideal polymerization of the resin material, the specimens were stored in a heating cabinet of 23 °C for 24 h. The cement gaps between the two components were measured, with values from 70 to 120 µm.

#### Simulated aging

All fixed samples underwent artificial thermal loading to simulate the moist atmosphere and the different temperatures in the oral cavity. With temperatures of 5 °C and 55 °C, a total of 10,000 thermal cycles were conducted [21–24]. Test specimens were immersed for 30 s in a temperature bath at each temperature within each cycle and were then exposed to room air for 10 s during transportation to the other bath. This procedure was conducted automatically. The specimens were returned to the heating cabinet at 23 °C for 24 h after the thermal loading process.

#### Retention force

**Fig. 4:**
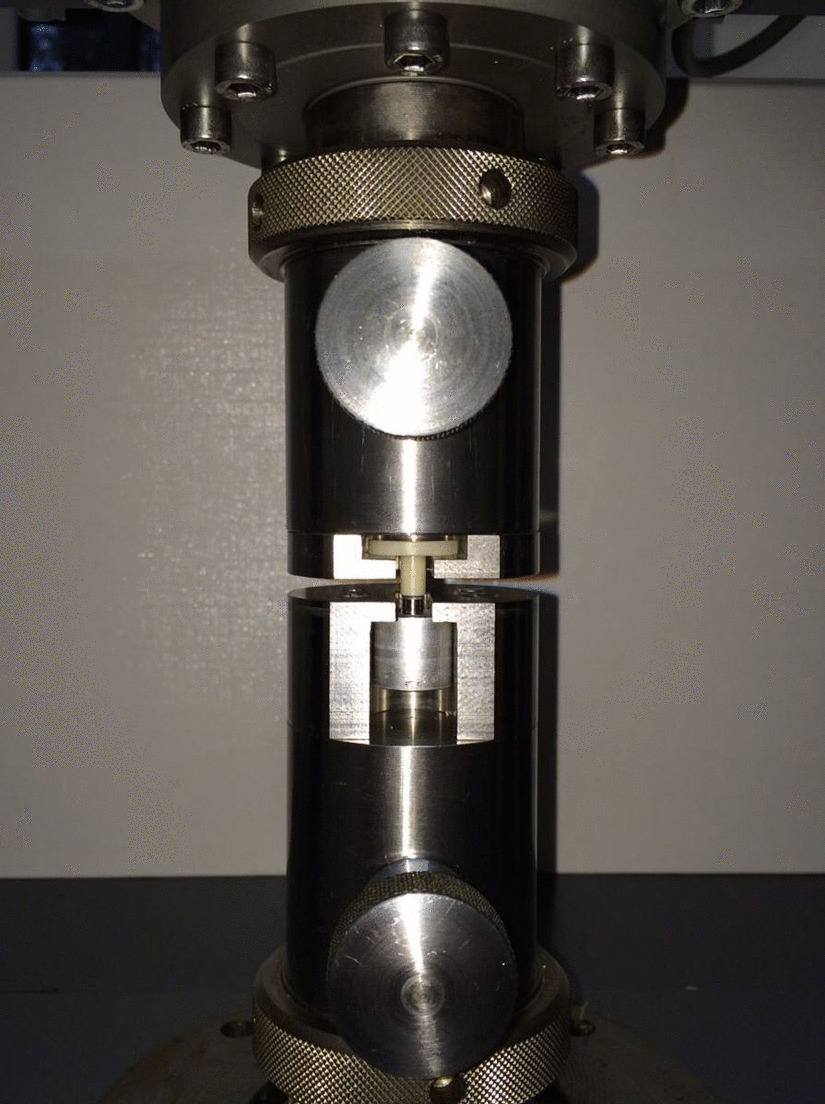
Test specimen installed into a universal test machine by using a special jig for pull-off testing

In the next step, all specimens were separated into ceramic coping and coated or uncoated base by using the pull-off test. For this procedure, the specimens were located into a special jig (Fig. 4) which was mounted in a universal test machine (Type 20 K, UTS Testsystems, Ulm-Einsingen, Germany). The design of the extractor tool ensured that the specimens were stably and reproducibly sited. The universal test machine moved vertically at a crosshead speed of 1 mm/min until the build-ups were completely separated from the titanium parts. The values for crosshead displacement and load were recorded (Programm Phoenix, UTS Testsysteme) during the loading, and the evaluated maximum force was defined as the retention force between the components.

### Fractographical analysis

After the retention force tests, fracture patterns of components were investigated with a reflected light microscope (M3Z, Wild, Heerbrug, Switzerland), with respect to surface quality, morphology, defects and reasons for failure. Typical patterns were recorded using a connected digital camera (progress C12 plus, Jenoptik, Jena, Germany). The residues of fixation composite on the surface areas of bases and copings were analyzed and classified into three different failure groups: residues only on the uncoated or coated titanium base, residues only on the ceramic build-ups, and residues on both parts. Representative fracture pattern of each class were analyzed in a scanning electron microscope (Philipps SEM 505, Philips, Eindhoven, the Netherlands).

### Statistical analysis

Statistical analysis was conducted using SPSS for Windows, version 19.0 (IBM, Ehringen, Germany). The normal distribution of data and homogeneity of variance were verified using the Kolmogorov–Smirnov and Levene tests, respectively. The arithmetic mean, the minimum, the maximum and the standard deviation were estimated for the measured retention forces in the pull-off test. The influence of surface pretreatments on the retention force between both parts were checked by two-way analysis of variance (ANOVA), with the level of significance set at 0.05. For the comparison of the different groups, the post-hoc Scheffé test was used.

## Results

### Retention force

The results of the retention forces were compared for the different surface treatments of the abutment. In Table 2, all treatment groups are listed. There were significant differences between titanium nitride coated and standardized uncoated titanium bases with respect to the retention force (two-way ANOVA, *p* < 0.05). For the titanium nitride coated bases of group D, a significant increase in retention force was observed, with 529 N in comparison to a force of 319 N for conventional titanium bases of group A (post-hoc Scheffé, *p* < 0.001) without any chemical pretreatment. On the other hand, the titanium nitride coating did not influence retention force after pretreatment with primer (groups E vs B and F vs. C). Moreover, treatment with primer alone did not influence the retention force (groups A vs. B, A vs. C, D vs. E, D vs. F).

**Table 2:** Pull-off forces for different test groups

Pull-off forces (N)				
Groups	Arithmetic mean	Minimum	Maximum	Standard deviation
A	319^b^	218	482	96
B	499^a^	340	757	130
C	599^a^	370	859	174
D	529^a^	462	595	42
E	477^a^	441	530	31
F	524^a^	462	652	52

Means with standard deviations, and medians with ranges are given. Values with the same superscript number do not differ with statistical significance (post-hoc, Scheffe, *p* ˂ 0.05)

### Analysis of fracture patterns

**Fig. 5:**
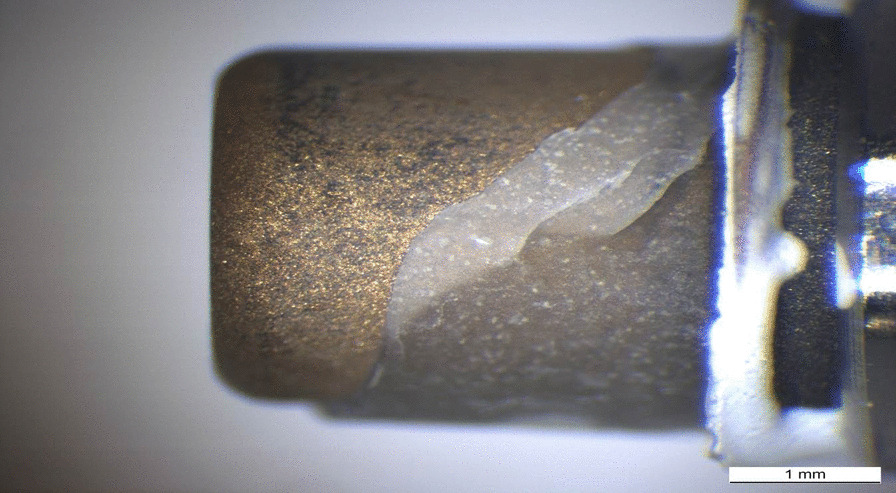
Typical example of a mixed fracture pattern. The titanium nitride surface is only partially covered with residues of the fixation resin

**Fig. 6:**
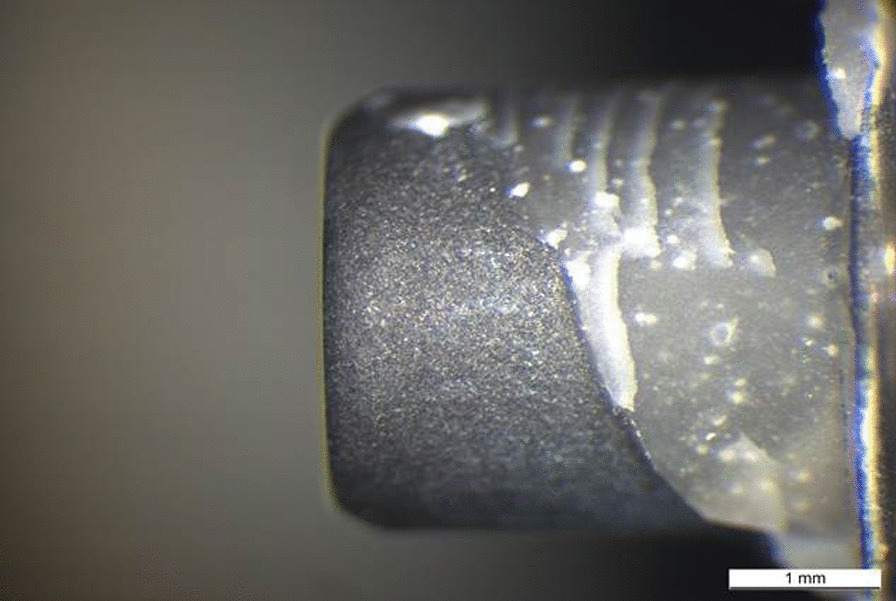
Typical example of a mixed fracture pattern. The pure titanium surface is only partially covered with residues of the fixation resin

**Fig. 7:**
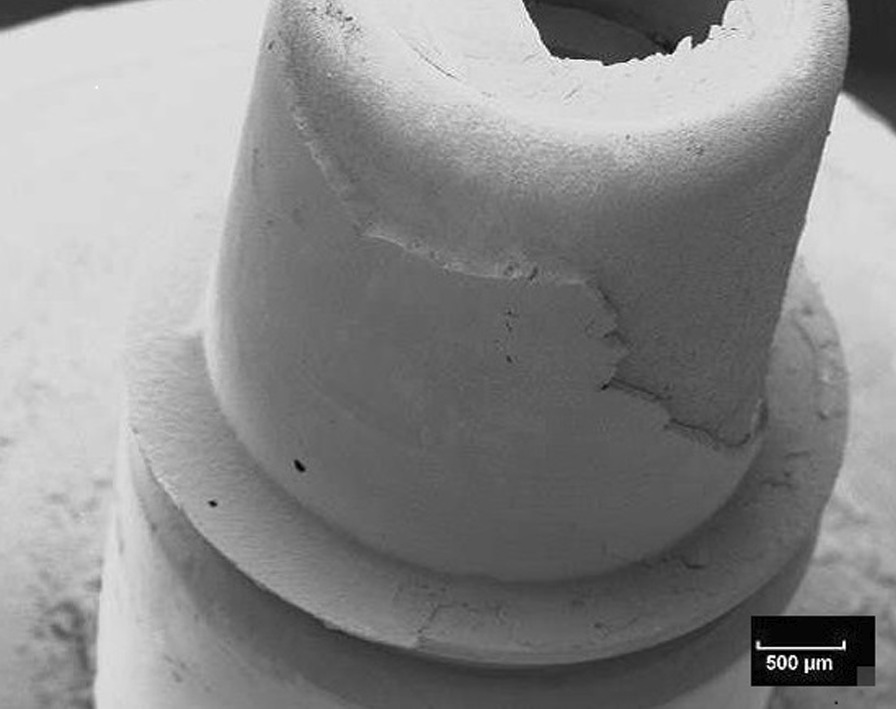
Scanning electron microscopic view of the titanium nitride base of the specimen shown in Fig. 5 (10kv, × 20, 50 nm). The adhesion of the base is covered by a homogenous layer of the fixation resin

**Fig. 8:**
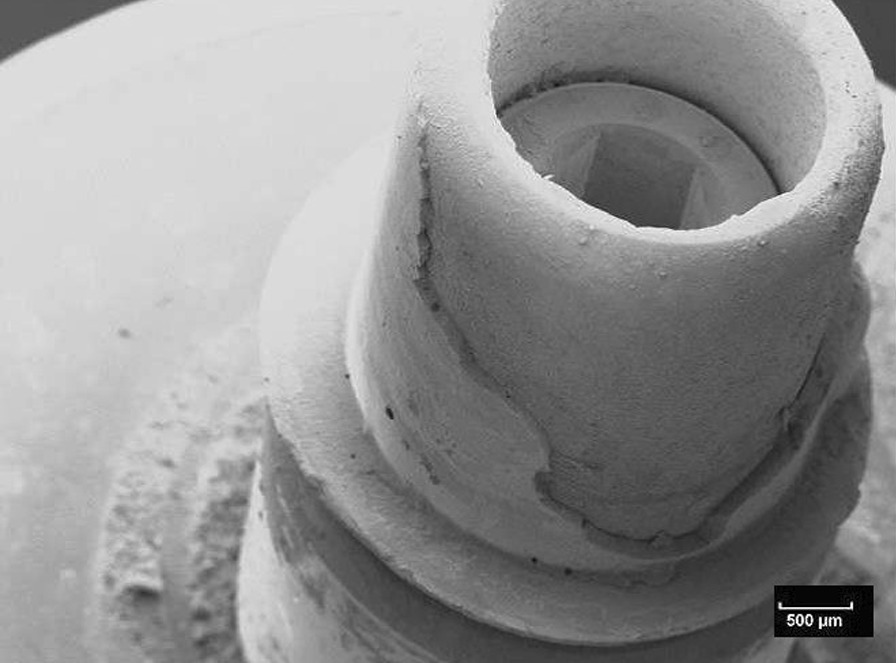
Scanning electron microscopic view of the titanium base of the specimen shown in Fig. 6 (10 kV, × 20, 50 nm). The adhesion of the base is covered by a homogenous layer of the fixation resin

**Fig. 9:**
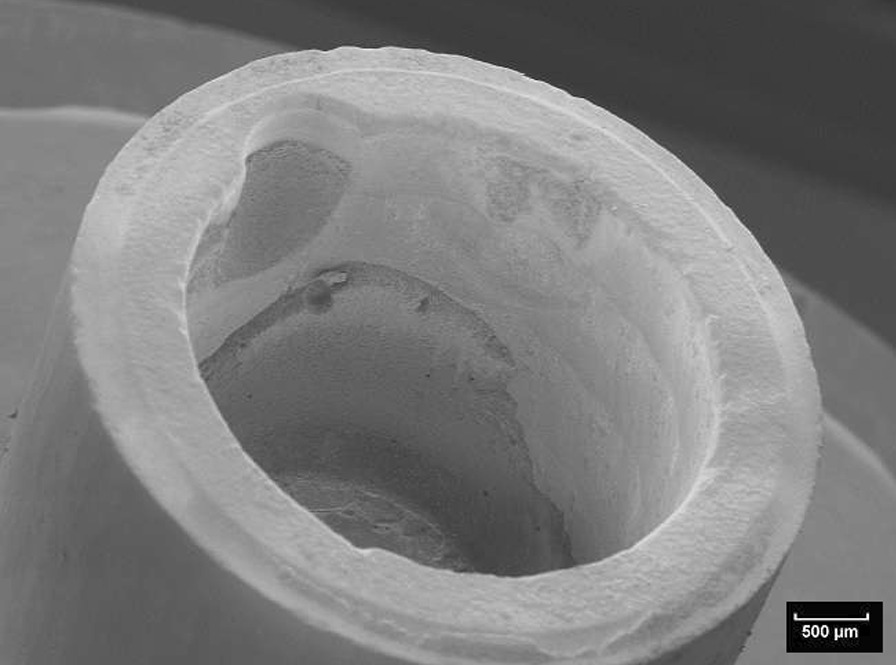
Scanning electron microscopic view of the ceramic buildup of the specimen shown in Fig. 7 (10kv, × 20, 50 nm). The adhesion of the build-up is covered by a homogenous layer of the fixation resin

**Fig. 10:**
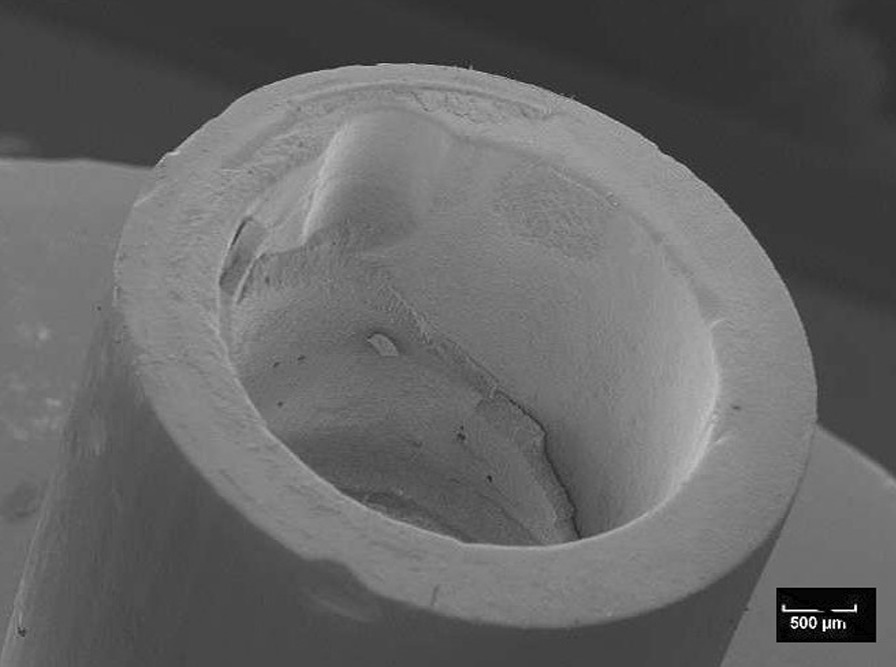
Scanning electron microscopic view of the ceramic buildup of the specimen shown in Fig. 8 (10 kv, × 20, 50 nm). The adhesion of the build-up is covered by a homogenous layer of the fixation resin

Patterns of the fracture surfaces were documented with digital photography (ProgRes C12 plus, Jenoptik, Jena, Germany). All test specimens showed mixed fracture patterns, where the bases and build-ups were coated by residues of fixation luting agent (Figs. 5, 6, 7, 8, 9, 10). The titanium nitride coated specimens did not differ from the uncoated bases in their fracture pattern analysis.

## Discussion

There have been only a few publications on retention forces in two-part abutments. Moreover, direct comparison with previous studies is difficult, as different luting agents, surface treatments and materials of the abutment components were used.

Two-part abutment systems for implant-supported prostheses consist of a ceramic build-up attached to bases composed of titanium—and the advantages of this approach have already been described [13, 20]. However, it is not known whether there are differences between coated bases and pure titanium bases with respect to the retention force to ceramic copings. Nevertheless, it is thought that the adhesive connection between the ceramic and the titanium or titanium coated parts may be a weak point in two-part abutment systems.

Only a few investigations have studied the forces of this adhesive bond and the influencing parameters [13, 14]. The main factor influencing retention between components of two-part abutment systems seems to be mechanical and chemical pretreatment. In an investigation of two-part abutment systems, Ebert et al. showed that pretreatment of zirconia copings with air-borne particles leads to an increase in retention force between the components [14]. That was also confirmed by von Maltzahn et al. in a further evaluation of the effect of surface modifications of two-part abutments [20]. In addition, Kurt et al. observed the highest retention forces with sandblasting, in comparison to other pretreatments [25]. In the present study, bases of pure titanium and titanium nitride coating were analyzed and compared. It was shown that—after airborne particle abrasion and coating with titanium nitride—no other treatment was necessary to optimize the retention force between the two components. Thus, it appears that with surface coating with titanium nitride, an increase in surface area as well as roughness is enough to create an adequately stable bond. Thus mechanical pretreatment with aluminum oxide powder appears to be an effective method to achieve higher forces between the bases and copings of two-part abutment systems.

Most published investigations have used aluminum oxide powder with a pressure of 0.25 MPa and an average particle size of 110 µm [26–28]. In the current study, a pressure of 0.2 MPa was applied to imitate a procedure which would be more effective in inhibiting detrimental microcracks at the ceramic surfaces [29, 30]. Not only airborne-particle abrasion, but also laser treatment may enhance bond strength [31]. The positive effect of surface treatment with a laser was confirmed by Gaggl et al. [32].

Furthermore, the influence of chemical or tribochemical surface modification was also investigated in the current study. Different surface conditioning techniques influenced the retention force of components of two-part abutments (Table 1). The highest bond strength was found for pure titanium bases with the use of the universal primer on both surfaces. On the other hand, in the group of coated bases, the use of this activating primer on both components only gave the second highest retention force. Other coated test specimens were pretreated with airborne-particle abrasion and metal primer on the bases and reached the lowest value for the retention force (477 N). In contrast to our results, Komine et al. examined the effect of different surface activating agents on the adhesive connection of resin-based materials to ceramic. They used the same metal primer and compared the maximum shear bond strength before and after thermal aging with alternative preconditioning processes. They found the highest strengths when the metal primer was applied both before and after aging [33]. Also Kern et al. analyzed the adhesion of Clearfil Ceramic Primer and Alloy Primer to ceramic especially zirconia and recorded the highest values of bond strength with the use of 10-methacryloyloxy-decyldihydrogenphosphate monomer containing (MDP) primers [34]. They suggested that this was due to linkage between functional primer monomers and metal ions on ceramic surfaces. Nevertheless, these functional monomers of the primers do not seem to lead to any increase in the adhesive bond of titanium nitride coated surfaces as used in the present study. This suggests that the adhesion of the phosphoric acid group of MDP-containing primers with the metal atoms of coating is not stable enough to maximize retention forces. The limited effect of the MDP-containing primers on the coated titanium surfaces could also be associated with the coating itself or the coating process, in which the normal oxide layer of the titanium is changed. This could make chemical bonding via oxygen to MDP monomers more difficult, since the titanium nitride contains nitrogen compounds. It could be assumed that the irregularities on the surface of the titanium nitride coatings—as evaluated by Tanaka et al. with electron probe microanalyses—are sufficient to form a retentive bond with the resin agent without chemical pretreatment. In addition, further investigations of the surface properties would be necessary to verify these assumptions [35].

After the pretreatment and fixation of components, all specimens underwent artificial aging by thermocycling between + 5 and + 55 °C for 10,000 cycles. The holding time of the samples per water bath was 30 s. The alternating time between the two baths was 10 s. The change between the baths was conducted automatically. In the literature, data on the temperature variations in the oral cavity e.g. during food intake, can differ between − 2 and + 80 °C [36, 37]. The effect of these thermal variations on dental restorations is between + 5 and + 55 °C [38–40]. Since ceramics are highly brittle, they are susceptible to cracking under stress caused by heat stress. For this reason, values of + 5 °C and + 55 °C were typically used for thermal aging.

The fracture patterns investigation showed mixed fracture patterns for the entire test specimens. These mixed fracture patterns were characterized by the resin residues on both the ceramic and titanium or titanium nitride surfaces. The analysis indicates that the connection between ceramic surface and the luting agent is similar for titanium and titanium nitride surfaces. Perhaps this is because mechanical and/or chemical pretreatment always tends to maximize bond strengths. Similar results were reported in an investigation of Inokoshi et al. They also concluded that mechanical and chemical pretreatment of surfaces influenced the adhesive connection between resin-based materials and dental ceramics, especially zirconia [41].

The findings of the current research demonstrated that techniques for surface modifications can lead to an increase in the retention forces of titanium nitride coated bases of two-part abutments. Although the test specimens were examined in vitro, the results can be transferred to the conditions of the oral cavity, if we take into account that only an approximate situation has been established. Cyclic forces of the oral cavity were not investigated with respect to the retention forces of specimen components, but masticatory forces may influence the durability of resin bonds. Therefore, the outcome of the current laboratory investigation is subject to the limitation that the analysis did not cover all conditions of the oral environment influencing the resin agents. But resins and surface pretreatments used on coated and uncoated bases of two part abutment systems provided enough evidence for clinical use. Nevertheless more studies are required to evaluate the best possible bonding connection between components of two-part abutments systems.

## Conclusions

Several conclusions can be drawn from this in vitro study. Firstly, mechanical pretreatment of the titanium nitride coated surfaces can significantly improve the adhesive connection between the components of two-part abutments. On the other hand, chemical preconditioning with silane coupling agents containing functional monomers has no effect on the retention force of titanium nitride coated bases. Consequently our initial hypothesis was confirmed that titanium nitride-coated bases gave comparable or higher values for the adhesive connection of the parts of two-part abutment systems. Thus, titanium nitride-coated bases seemed to be an alternative for two-part abutments with clinically dependable retention force to ceramic copings. But in practice, many more factors must be considered that might influence the selection of the method of bonding—including the ease of processing, production time, cost effectiveness, and acceptance by dentist and dental technicians. In addition, further studies—especially clinical studies- on the retention force should be carried out to investigate other coating options besides the titanium nitride coating, as used in the present analysis. Further pretreatments and materials should also be added and compared.

## Data Availability

The datasets used and/or analyzed during the current study available from the corresponding author on reasonable request.

## Abbreviations

CAD: computer-aided design
CAM: computer-aided manufacturing
TiN: titanium nitride
Y-TZP: yttrium stabilized tetragonal zirconia ceramic
MDP: 10-methacryloyloxydecyl dihydrogenphosphate
